# Complications and Pathophysiology of COVID-19 in the Nervous System

**DOI:** 10.3389/fneur.2020.573421

**Published:** 2020-12-04

**Authors:** Haiyang Yu, Tong Sun, Juan Feng

**Affiliations:** ^1^Department of Neurology, Shengjing Hospital of China Medical University, Shenyang, China; ^2^Department of Pediatrics, Shengjing Hospital of China Medical University, Shenyang, China

**Keywords:** coronavirus, COVID-19, SARS-CoV-2, nervous system, complication, pathophysiology

## Abstract

The coronavirus disease (COVID-19) pandemic, caused by the severe acute respiratory syndrome coronavirus 2 (SARS-CoV-2), has become a global public health threat. Majority of the patients with COVID-19 have fever, cough, and fatigue. Critically ill patients can develop dyspnea and acute respiratory distress syndrome. In addition to respiratory symptoms, neurological damage also occurs in some patients. However, the mechanisms by which SARS-CoV-2 invades the nervous system have not been elucidated yet. In order to provide some reference for designing optimal therapeutic strategies, we have discussed the complications and potential mechanisms of COVID-19 in the nervous system in this review.

## Introduction

Coronavirus disease (COVID-19), caused by the severe acute respiratory syndrome coronavirus 2 (SARS-CoV-2), has spread worldwide (1). Because of the rapid spread of the virus and a sharp increase in the number of confirmed cases, the World Health Organization declared it a pandemic on March 11, 2020.

To date, seven human coronaviruses have been known to infect humans, three of which have resulted in epidemics (2, 3). First, the severe acute respiratory syndrome (SARS), caused by the SARS coronavirus (SARS-CoV), started in Asia and then spread across the world in 2002 and 2003 (4). This was followed by the Middle East respiratory syndrome (MERS), caused by the MERS coronavirus (MERS-CoV), with a high mortality rate in 2012 (5). The current COVID-19 pandemic is the third and is still attracting global attention.

SARS-CoV-2 is considered a member of the beta coronaviruses (β-CoVs) family, which also contains SARS-CoV and MERS-CoV (6). The CoV family consists of enveloped, positive-sense single-stranded RNA viruses. They are spherical or oval in shape with large glycoprotein spikes on the surface and display a typical crown-like shape on negative staining when observed by electron microscopy. The CoV family is divided into four subfamilies genotypically and serologically, namely, α, β, γ, and δ-CoVs. Among them, α- and β-CoVs can cause human infection (7). Generally, all human CoVs are zoonotic, and bats are the most likely natural hosts of CoVs (8). Moreover, before CoVs infect humans, they need intermediate animal hosts of CoVs, which are civet cats and dromedary camels for SARS-CoV and MERS-CoV, respectively (9, 10). The discovery of pangolin CoVs and their similarity to SARS-CoV-2 indicate that pangolins may be the possible intermediate hosts for SARS-CoV-2 (11).

Currently, patients with COVID-19 are the main sources of infection. However, asymptomatic carriers have also been proven to excrete the virus and may be potential sources of infection (12). Respiratory droplets and close contact are regarded as the main transmission routes. It has been reported that SARS-CoV-2 may be isolated from the feces and urine. Therefore, attention should be paid to aerosol or contact transmission as a result of environmental contamination by the feces and urine of infected individuals (13). Since the population is generally susceptible to SARS-CoV-2, COVID-19 has spread rapidly worldwide. Several retrospective cohort studies have suggested that patients who are older in age and have hypertension, high lactate dehydrogenase level, high Sequential Organ Failure Assessment score, d-dimer >1 μg/mL, and cancer were more likely to show deterioration and develop severe illness with a poor prognosis (14, 15). In addition, male sex, severe illness, expectoration, muscle ache, and decreased albumin were found to be independent risk factors that could influence the clinical course of COVID-19 patients. Furthermore, severely ill men with a heart injury, hyperglycemia, and high-dose corticosteroid use may have a higher risk of death (16, 17). Smoking may be a high-risk factor for infection, too (18).

**Figure 1:**
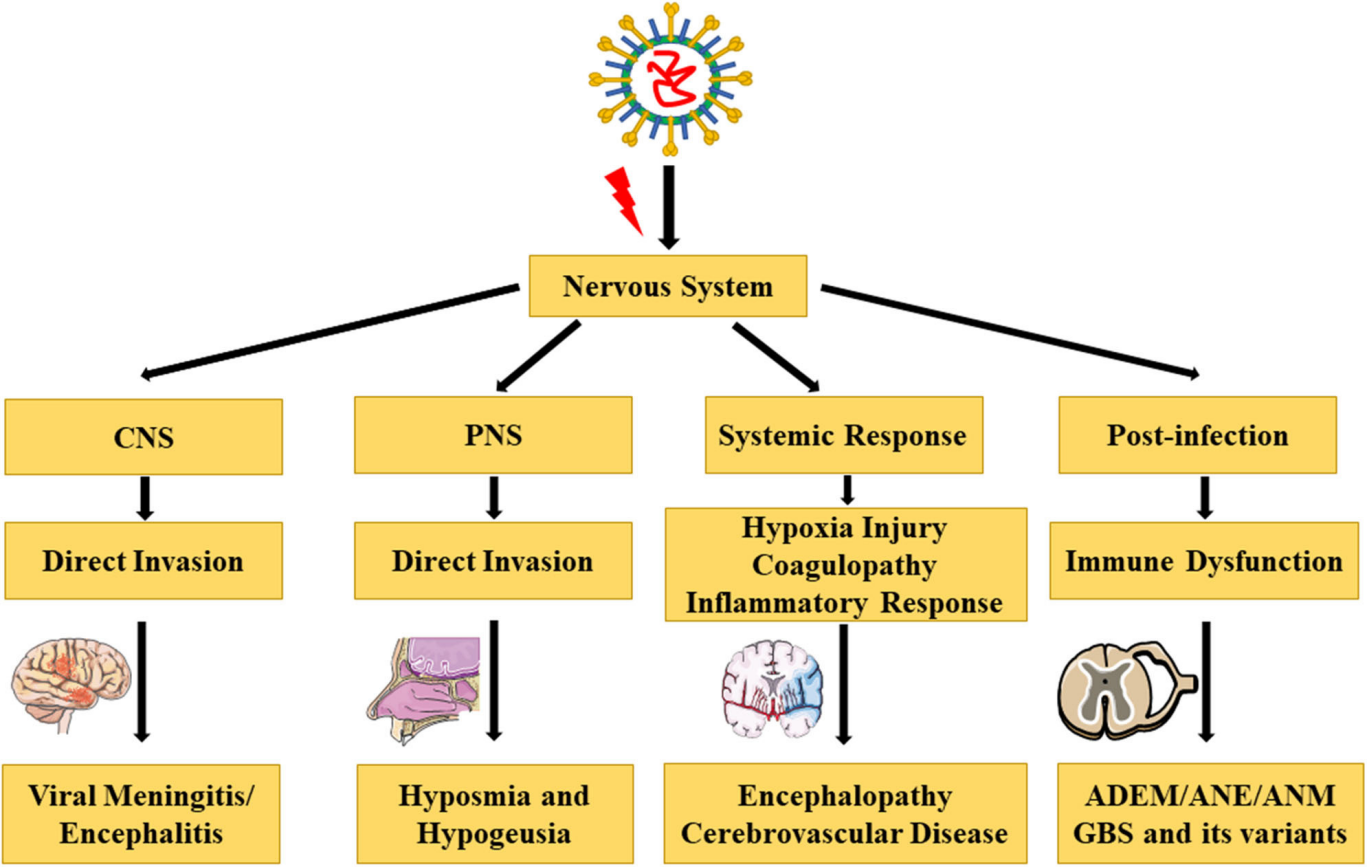
Complications and pathophysiology of COVID-19 in the nervous system [The illustrations are provided by Servier Medical Art (https://smart.servier.com/) licensed under a Creative Commons Attribution 3.0 Unported License].

COVID-19 mainly manifests with fever, dry cough, and fatigue. Few patients have symptoms of a stuffy or runny nose, headache, myalgia, and diarrhea. Most of the critically ill patients develop dyspnea or hypoxia 1 week after the onset of illness. With rapid progression of the disease, acute respiratory distress syndrome (ARDS), septic shock, and metabolic acidosis can develop (19). According to the pathological findings from limited autopsies and biopsies, SARS-CoV-2 can invade multiple tissues and organs in addition to the lung, such as the spleen, liver, heart, kidney, and brain (20, 21). Autopsy results have revealed that SARS-CoV-2 RNA could be detected in the brain tissue in 36.4% of fetal cases, which indicates the neurotropism and potential for invasion of SARS-CoV-2 in the brain (22). Recently, Zhang et al. were the first to prove that SARS-CoV-2 could directly infect induced pluripotent stem cells–derived human neural progenitor cells, and extensive viral replication and viral particles were detected in the neurospheres and brain organoids with SARS-CoV-2 infection. Additionally, they showed that SARS-CoV-2 could productively infect the human brain (23). A retrospective case series demonstrated that the neurological symptoms include central nervous system (CNS) symptoms or diseases (headache, dizziness, impaired consciousness, ataxia, acute cerebrovascular disease, and epilepsy), peripheral nervous system (PNS) symptoms (hyposmia, hypogeusia, hypopsia, and neuralgia), and skeletal muscle symptoms (24). In this review, we have discussed the principal COVID-19–related complications and pathophysiology in the nervous system (Table 1, Figure 1).

**Table 1:** Clinical manifestations and auxiliary examination findings of COVID-19 related complications in the nervous system.

**Complication**	**Clinical manifestations**	**Auxiliary examination**	**References**
**COVID-19–RELATED COMPLICATIONS IN THE NERVOUS SYSTEM**			
Viral meningitis/encephalitis	Headache Altered mental status, meningeal irritation signs	CSF: positive PCR assay for SARS-CoV-2 Increased lymphocytes and proteins MRI: FLAIR hyperintensity EEG: slowing	(25–30)
Acute disseminated encephalomyelitis (ADEM)	Multifocal deficits	MRI: FLAIR hyperintensity, multifocal demyelinating lesions Autopsy: ADEM-like appearance in the subcortical white matter	(31–33)
Encephalopathy	Headache, altered mental status	CSF: negative PCR assay for SARS-CoV-2 EEG: diffuse slowing	(34–36)
Acute necrotizing encephalopathy (ANE)	Altered mental status	CT: hypoattenuation MRI: T2 FLAIR hyperintensity with internal hemorrhage	(37)
Cerebrovascular disease	Sensory or motor dysfunction	CT/MRI: ischemic or hemorrhagic change	(38–40)
Epilepsy	Seizures	CSF: negative PCR assay for SARS-CoV-2 EEG: semirhythmic, irregular, high-amplitude delta waves	(41–43)
Acute myelitis	Flaccid paralysis, hypesthesia Urinary and bowel dysfunction	MRI: T2 hyperintensity	(31, 44, 45)
Hyposmia and hypogeusia	Loss of a sense of smell and taste	Questionnaire-based survey Cross-sectional study	(46–49)
Guillain-Barré syndrome (GBS)	Flaccid paralysis	CSF: negative PCR assay for SARS-CoV-2 MRI: enhancement of affected nerve roots EMG: decreased recruitment	(50–53)
Miller Fisher syndrome (MSF)	Ophthalmoplegia, ataxia, and areflexia	MRI: relative enlargement, T2 hyperintensity, and enhancement of the affected CN Anti-GD1b antibody positive	(54, 55)

*COVID-19, coronavirus disease; SARS-CoV-2, severe acute respiratory syndrome coronavirus 2; CSF, cerebrospinal fluid; CT, computed tomography; MRI, magnetic resonance imaging; PCR, polymerase chain reaction; EEG, electroencephalography; EMG, electromyography*.

## COVID-19–Related Complications in the Nervous System

### COVID-19–Related Complications in CNS

#### Viral Meningitis/Encephalitis

Viral infection can cause both meningitis and encephalitis, which are inflammation of the meninges and brain parenchyma, respectively (56). A man with COVID-19 exhibited meningeal irritation signs (nuchal rigidity, Kernig sign, and Brudzinski sign), along with positive extensor plantar response. After excluding bacterial or tuberculous infections of the CNS, SARS-CoV-2 encephalitis was diagnosed. However, SARS-CoV-2 in the CSF specimen was negative, which might be due to the extremely low titer of the virus in the CSF, or due to the lack of a standardized test for SARS-CoV-2 detection in the CSF (25). In Japan, a case report described the first patient who was sent to the emergency department because of a convulsion accompanied by unconsciousness and who was subsequently diagnosed with aseptic encephalitis with the SARS-CoV-2 RNA detected in the CSF (26). Similarly, in Los Angeles, a young woman with COVID-19 showed symptoms of meningoencephalitis without respiratory failure, and SARS-CoV-2 was found to be positive in the CSF by reverse transcription–polymerase chain reaction (PCR) (27, 28). In addition, two patients with acute meningoencephalitis concomitant with SARS-CoV-2 infection were reported in Switzerland, and a case of rhombencephalitis was reported as a rare complication of acute COVID-19 infection in the United Kingdom (29, 30).

#### Acute Disseminated Encephalomyelitis

Acute disseminated encephalomyelitis (ADEM) is an idiopathic CNS demyelinating disease, which is often postviral and is common in children, although it can occur at any age. The first case of COVID-19–related ADEM was reported in a 40-year-old woman, and magnetic resonance imaging (MRI) revealed fluid-attenuated inversion recovery (FLAIR) hyperintensities in the subcortical and deep white matter (31). A 51-year-old woman developed a coma and impaired unilateral oculocephalic response weeks after a SARS-CoV-2 infection. Her MRI demonstrated acute multifocal demyelinating lesions, and the clinical examination and CSF analysis were consistent with an acute demyelinating event (32). Furthermore, the autopsy of a 71-year-old patient diagnosed with COVID-19 showed scattered clusters of macrophages, axonal injury, and a perivascular ADEM-like appearance in the subcortical white matter (33).

#### Encephalopathy

In a retrospective study of 113 deceased patients with COVID-19, Chen et al. reported that 20% of cases demonstrated hypoxic encephalopathy, which is a higher proportion when compared with recovered patients (34). Elderly patients with chronic conditions are more susceptible to COVID-19, and patients with prior neurological conditions and acute respiratory symptoms are at an increased risk of encephalopathy. A 74-year-old man presented with symptoms of encephalopathy including headache and altered mental status and was diagnosed with COVID-19. As the CSF examination was normal, the neurological symptoms were not due to meningitis or encephalitis (35). Similarly, a 72-year-old man also presented with COVID-19 infection and encephalopathy. Subsequent CSF studies showed no evidence of a CNS infection. However, electroencephalography (EEG) revealed diffuse slowing consistent with an encephalopathy (36).

#### Acute Necrotizing Encephalopathy

Notably, Poyiadji et al. reported the first case of SARS-CoV-2 infection associated with acute hemorrhagic necrotizing encephalopathy. Acute necrotizing encephalopathy (ANE) is a rare encephalopathy that has been associated with influenza or other viral infections, which results in breakdown of the blood–brain barrier (BBB), without direct viral invasion or parainfectious demyelination. Imaging features are characterized by multifocal symmetric lesions in the thalami. However, the brain stem, cerebral white matter, and cerebellum may also be affected (37).

#### Cerebrovascular Disease

Cerebrovascular disease has been associated with an increased disease severity in patients with COVID-19 (38), and is also emerging as an important complication of COVID-19. Mao et al. reported that patients with a severe infection were more likely to develop neurological manifestations later in the course of the illness, especially acute cerebrovascular disease and impaired consciousness (24). However, Avula et al. reported four patients with PCR-confirmed SARS-CoV-2 infection, who presented with an acute ischemic stroke, and all four cases presented with a cerebrovascular accident in a relatively early stage of the illness (39). Moreover, two cases of cerebral hemorrhage have been reported by Al Saiegh et al. (40).

#### Epilepsy

In Italy, Vollono et al. reported a patient with COVID-19 whose primary symptom was a focal status epilepticus in the context of a predisposing but well-controlled postencephalitic epilepsy. Although the patient exhibited no fever or respiratory symptoms, the diagnostic hypothesis of a SARS-CoV-2 infection could be made on the basis of the worsening or recurrence of paroxysmal neurological events. Therefore, it is possible to hypothesize that SARS-CoV-2 could trigger seizures through a neurotropic pathogenic mechanism (41). However, a man without any history of epilepsy developed multiple episodes of seizures after infection with SARS-CoV-2 (42). In addition, an infant with both COVID-19 and rhinovirus infections also presented with seizures, although no changes were observed in the EEG (43).

#### Spinal Cord Injury

Besides involvement of the brain, SARS-CoV-2 can also damage the spinal cord. On admission to hospital, a 66-year-old man with COVID-19 presented with acute flaccid paralysis of bilateral lower limbs and urinary and bowel incontinence. He was diagnosed with postinfectious acute myelitis (31). In addition, a case of multifocal transverse myelitis has also been reported. After recovering from COVID-19, a 60-year-old man developed progressive weakness of the lower limbs and bladder dysfunction. Two days later, he showed hypesthesia below the Th9 level and a moderate spastic paraparesis. MRI of the spine revealed T2 signal hyperintensity of the thoracic spinal cord at the Th9 level, suggesting acute transverse myelitis (44). Moreover, a 69-year-old woman was diagnosed with acute necrotizing myelitis (ANM) based on the clinical symptoms and MRI manifestations (45).

### COVID-19–Related Complications in the PNS

#### Hyposmia and Hypogeusia

In many countries, patients with COVID-19 have reported a loss of the sense of smell and taste. In South Korea, hyposmia was quite frequent among patients with mild COVID-19, and accompanying symptoms such as hypogeusia appeared in most of the patients with hyposmia (46). In Italy, a cross-sectional survey proved that olfactory and taste disorders were present in the early stages of the SARS-CoV-2 infection (47). Furthermore, researchers from France noticed that hyposmia and hypogeusia were reported during the early phase of the COVID-19 outbreak, and they investigated the utility of these symptoms for the early diagnosis of COVID-19 (48). Therefore, for patients with mild and inconspicuous symptoms and those in the early phase of illness, social distancing should be strongly implemented to prevent disease transmission (49).

#### Guillain-Barré Syndrome and Its Variants

Guillain-Barré syndrome (GBS) is an autoimmune-induced neuropathy, which mainly targets the peripheral nerves and their spinal roots. It is usually caused by an infection or immune stimulation that induces an aberrant autoimmune response (57). Several cases of GBS have been reported in patients with COVID-19 (50–53). However, additional epidemiological data are necessary to support a causal relationship between GBS and COVID-19.

Miller Fisher syndrome (MSF) is a variant of GBS and is an acute peripheral neuropathy that is manifested with a triad of ophthalmoplegia, ataxia, and areflexia. A 36-year-old man infected with SARS-CoV-2 showed diplopia due to cranial nerve (CN) III palsy. MRI revealed relative enlargement, T2 hyperintensity, and enhancement of the affected CN III (54). Similarly, a 50-year-old man presented with the triad of MSF and was positive for one of the antibodies to gangliosides (GD1b antibodies) (55).

## COVID-19–Related Pathophysiology in the Nervous System

### Direct Invasion

CoVs can invade the CNS through direct hematogenous and neural propagation (58). In case of hematogenous dissemination, CoVs in the airways can pass through the epithelial barrier reaching the blood or lymph circulation and then propagate toward the CNS. The BBB is composed of endothelial cells that interact with pericytes, astrocytes, microglia, and neurons in the neurovascular unit and regulate the permeability of the BBB and consequently maintain the integrity of the CNS (59). The diameter of the SARS-CoV-2 is 60–140 nm, making it easy for the virus to bypass the BBB and gain entry into the CNS (60).

The other route for invasion by the CoVs into the CNS is through neural dissemination, possibly by the polarization of neurons. This transport can be retrograde or antegrade and is facilitated by dynein and kinesin (61). Hyposmia in patients with COVID-19 may due to “conductive” loss or “neural” loss (62). Rapid recovery of normal olfaction in patients suggests a “conductive” loss, called olfactory cleft syndrome, and is associated with mucosal obstruction of the olfactory cleft (63). Fodoulian et al. showed that angiotensin-converting enzyme 2 (ACE2) and transmembrane serine protease 2 are predominantly expressed in the non-neuronal cells of the olfactory epithelium and olfactory bulb in both mice and humans (64). Recently, neuropilin-1 (NRP1) was found to be expressed in the olfactory epithelium. Therefore, both ACE2 and NRP1 may be involved in the transmission of SARS-CoV-2 from the olfactory nerves to the CNS (65). Additionally, Wang et al. reported that SARS-CoV-2 could infect mature and immature olfactory neurons along with the supporting sustentacular cells in hamsters, and this may contribute to the unique olfactory dysfunction of COVID-19 (66). Although the olfactory bulb is important in early virologic control, several studies have proven that the olfactory route is an important pathway for viral entry into the CNS (67).

### Angiotensin-Converting Enzyme 2

Similar to the SARS-CoV, SARS-CoV-2 can infect humans by targeting ACE2 via its spike protein. SARS-CoV-2 displays more specificity in recognizing ACE2 and has a stronger binding affinity with ACE2 due to the presence of a receptor-binding domain (68). ACE2 has multiple physiological roles, such as cell proliferation, blood pressure regulation, and inflammatory response. ACE2 is widely expressed in the lungs, kidneys, guts, cardiovascular system, and even the CNS, indicating that SARS-CoV-2 may affect multiple organs and systems (69). In the brain, ACE2 is expressed in the neurons, astroglia cells, microglia cells, and endothelial cells (70). ACE2 is a negative regulator of the renin–angiotensin system, all components of which are present in the brain (71). Depletion of ACE2 increases the expression of angiotensin II, leading to vasoconstriction, sodium and water retention, elevated blood pressure, proinflammatory, and procoagulation effects (72). As SARS-CoV-2 binds to ACE2, some patients may demonstrate unusually high blood pressure and an increased risk of acute cerebrovascular disease. Given that SARS-CoV-2 targets ACE2 as the receptor, preventing the binding of SARS-CoV-2 with ACE2 may be a potential therapeutic strategy for preventing damage to multiple organs (73).

### Hypoxic Injury

Severe patients with COVID-19 may develop ARDS, characterized by a serious shortness of breath and hypoxemia (19). The neuro-invasive potential of SARS-CoV2 may play a role in the respiratory failure seen in patients with COVID-19 (74). Hypoxia can cause a series of pathological changes in multiple organs. Pathological findings of COVID-19 associated with ARDS show pulmonary edema with hyaline membrane formation, which can lead to gas exchange disorders and hypoxia in the CNS (21). Hypoxia induces an excessive accumulation of anaerobic metabolites in the mitochondria and acid metabolites in brain, leading to edema of the brain cells and obstruction of the cerebral blood flow (75). The guidelines for the diagnosis and treatment of COVID-19 (trial version 8 in Chinese) (Supplementary Material 1) described the pathological changes consisting of brain congestion, edema, and degeneration of a part of the neurons on autopsy, which are similar to those seen in infection with SARS (76). Thus, severe hypoxia may be a high-risk factor for hypoxic encephalopathy and ischemic stroke, causing serious damage to the nervous system.

### Coagulopathy

Zhang et al. reported that coagulopathy and antiphospholipid antibodies were present in three patients with COVID-19 (77). Moreover, a retrospective analysis revealed that abnormal coagulation results, including markedly elevated d-dimer and fibrin degradation product levels, as well as a longer prothrombin time and activated partial thromboplastin time, are associated with poor prognosis. Additionally, disseminated intravascular coagulation is more commonly associated with COVID-19 deaths (78). The hypercoagulability seen in patients with COVID-19 may predispose to a stroke (79). Consequently, anticoagulant treatment may decrease the mortality in severe COVID-19 patients with coagulopathy (80).

### Inflammatory Response

ARDS is the principal cause of death in patients with COVID-19 (19). One of the main mechanisms of ARDS is a cytokine storm, which is a deadly systemic inflammatory response, characterized by the release of large amounts of proinflammatory cytokines and chemokines by immune effector cells, including interleukin 2 (IL-2), IL-6, IL-7, IL-10, and IL-1β, as well as interferon γ, tumor necrosis factor α (TNF-α), GCSF, IP10, MCP1, and MIP1A (19, 81). Cytokine storm can induce an immune attack in the body, causing multiple organ failure and ARDS (82). Additionally, previous studies have shown that the coronavirus can induce proinflammatory cytokine signals from astrocytes and microglia cells, releasing a large amount of inflammatory factors such as IL-6, IL-12, IL-15, IL-1β, and TNF-α. This is also one of the pathophysiological processes responsible for CNS damage caused by inflammatory factors (83). Given the substantial increase in the proinflammatory cytokines, therapies such as plasma exchange and the IL-6 receptor-targeted monoclonal antibody are used to ameliorate the inflammatory response (84, 85). Furthermore, Lianhuaqingwen, a traditional Chinese medicine, has been proven to exert anti-inflammatory activity against SARS-CoV-2 *in vitro* (86).

### Immune Dysfunction

In severe patients with COVID-19, peripheral CD4^+^ T and CD8^+^ T cell numbers are significantly reduced, although are in a hyperactivated state. Moreover, there are high concentrations of proinflammatory CCR6^+^ T_h_17 in CD4^+^ T cells and cytotoxic granules in CD8^+^ T cells, suggesting that the overactivation of T cells is associated with immune injury in severe patients (21). In addition, a significant and progressive decrease in the lymphocytes is considered a sign of severity (87). Neutralizing antibodies in convalescent plasma were used to treat five critically ill patients with COVID-19 and ARDS, and their clinical status improved (88). However, Wang et al. found that severe patients had an increased immunoglobulin G (IgG) response and higher levels of IgG, especially anti–spike IgG (anti–S-IgG) neutralizing antibodies (89). This suggested that antibody-dependent enhancement (ADE) was present in SARS-CoV-2 infection, similar to what has been observed in multiple viral infections (90). ADE can promote cellular uptake of virus–antibody complexes (virus-anti–S-IgG) by interacting with the Fc receptor or other receptors, leading to enhanced invasion of the virus (91). Therefore, immune system can be activated by a viral infection, and activation of the immune cells in the brain may cause chronic inflammation and neurological damage (92). Furthermore, immune dysfunction after SARS-CoV-2 infection can result in a series of postinfectious diseases, such as ADEM, ANE, ANM, and GBS and its variants.

## Discussion

The pandemic of COVID-19 has become a global concern, and the respiratory system is not the only system involved in this disease. In this review, we discussed the main neurological complications and potential mechanisms of COVID-19. Neurological manifestations including hyposmia and hypogeusia may be the first symptoms of COVID-19 and may help in early detection, diagnosis, isolation, and treatment. With disease progression, more severe neurological symptoms may appear in critically ill patients, such as encephalitis, encephalopathy, and acute cerebrovascular disease. Nevertheless, the full clinical spectrum of neurological symptoms in patients with COVID-19 remains to be characterized. Neurologists should pay attention to these neurological manifestations and follow them up for possible neurological sequelae. It is exciting to see that several vaccines for COVID-19 are in clinical trials (NCT04398147; NCT04456595; NCT04466085; NCT04368728), although there are still no effective drugs to treat COVID-19 (93). Therefore, people across the world still need to make a huge effort to combat the COVID-19 pandemic.

## Author Contributions

JF conceived and revised this review. HY took charge of the original manuscript writing. TS drew the figure and table. All authors contributed to the article and approved the submitted version.

## Conflict of Interest

The authors declare that the research was conducted in the absence of any commercial or financial relationships that could be construed as a potential conflict of interest.

## Abbreviations

COVID-19: Coronavirus disease 2019
SARS-CoV-2: Severe acute respiratory syndrome coronavirus 2
ARDS: Acute respiratory distress syndrome
CNS: Central nervous system
PNS: Peripheral nervous system
ADEM: Acute disseminated encephalomyelitis
ANE: Acute necrotizing encephalopathy
ANM: Acute necrotizing myelitis
GBS: Guillain-Barré syndrome
MSF: Miller Fisher syndrome
BBB: Blood–brain barrier
PCR: Polymerase chain reaction
ACE2: angiotensin-converting enzyme 2.
