# Supplementary material for: Complications and Pathophysiology of COVID-19 in the Nervous System
Source: Front Neurol. 2020 Dec 4;11:573421. doi: 10.3389/fneur.2020.573421 (PMC7746805; doi:10.3389/fneur.2020.573421)
Supplement: Supplementary file 1 [file Data_Sheet_1.pdf]

新型冠状病毒肺炎诊疗方案
（试行第八版）

新型冠状病毒肺炎（新冠肺炎，COVID-19）为新发急性呼吸道传染病，目前已成为全球性重大的公共卫生事件。通过积极防控和救治，我国境内疫情基本得到控制，仅在个别地区出现局部暴发和少数境外输入病例。由于全球疫情仍在蔓延，且有可能较长时期存在，新冠肺炎在我国传播和扩散的风险也将持续存在。为进一步加强对该病的早发现、早报告、早隔离和早治疗，提高治愈率，降低病亡率，在总结我国新冠肺炎诊疗经验和参考世界卫生组织及其他国家诊疗指南基础上，我们对《新型冠状病毒肺炎诊疗方案（试行第七版）》进行修订，形成《新型冠状病毒肺炎诊疗方案（试行第八版）》。

一、病原学特点

新型冠状病毒（2019-nCoV）属于β属的冠状病毒，有包膜，颗粒呈圆形或椭圆形，直径 60～140nm。具有 5 个必需基因，分别针对核蛋白（N）、病毒包膜（E）、基质蛋白（M）和刺突蛋白（S）4 种结构蛋白及 RNA 依赖性的 RNA 聚合酶（RdRp）。核蛋白（N）包裹 RNA 基因组构成核衣壳，外面围绕着病毒包膜（E），病毒包膜包埋有基质蛋白（M）和刺突蛋白（S）等蛋白。刺突蛋白通过结合血管紧张素转化酶 2（ACE-2）进入细胞。体外分离培养时，新型冠状病毒 96 个小时左右即可在人呼吸道上皮细

胞内发现，而在 Vero E6 和 Huh-7 细胞系中分离培养约需 4～6 天。

冠状病毒对紫外线和热敏感，56℃ 30 分钟、乙醚、75%乙醇、含氯消毒剂、过氧乙酸和氯仿等脂溶剂均可有效灭活病毒，氯己定不能有效灭活病毒。

二、流行病学特点

（一）传染源。

传染源主要是新型冠状病毒感染的患者和无症状感染者，在潜伏期即有传染性，发病后 5 天内传染性较强。

（二）传播途径。

经呼吸道飞沫和密切接触传播是主要的传播途径。接触病毒污染的物品也可造成感染。

在相对封闭的环境中长时间暴露于高浓度气溶胶情况下存在经气溶胶传播的可能。

由于在粪便、尿液中可分离到新型冠状病毒，应注意其对环境污染造成接触传播或气溶胶传播。

（三）易感人群。

人群普遍易感。感染后或接种新型冠状病毒疫苗后可获得一定的免疫力，但持续时间尚不明确。

三、病理改变

以下为主要器官病理学改变和新型冠状病毒检测结果（不包括基础疾病病变）。

（一）肺脏。

肺脏呈不同程度的实变。实变区主要呈现弥漫性肺泡损伤

和渗出性肺泡炎。不同区域肺病变复杂多样，新旧交错。

肺泡腔内见浆液、纤维蛋白性渗出物及透明膜形成；渗出细胞主要为单核和巨噬细胞，可见多核巨细胞。II 型肺泡上皮细胞增生，部分细胞脱落。II 型肺泡上皮细胞和巨噬细胞内偶见包涵体。肺泡隔可见充血、水肿，单核和淋巴细胞浸润。少数肺泡过度充气、肺泡隔断裂或囊腔形成。肺内各级支气管黏膜部分上皮脱落，腔内可见渗出物和黏液。小支气管和细支气管易见黏液栓形成。可见肺血管炎、血栓形成（混合血栓、透明血栓）和血栓栓塞。肺组织易见灶性出血，可见出血性梗死、细菌和（或）真菌感染。病程较长的病例，可见肺泡腔渗出物机化（肉质变）和肺间质纤维化。

电镜下支气管黏膜上皮和 II 型肺泡上皮细胞胞质内可见冠状病毒颗粒。免疫组化染色显示部分支气管黏膜上皮、肺泡上皮细胞和巨噬细胞呈新型冠状病毒抗原免疫染色和核酸检测阳性。

（二）脾脏、肺门淋巴结和骨髓。

脾脏缩小。白髓萎缩，淋巴细胞数量减少、部分细胞坏死；红髓充血、灶性出血，脾脏内巨噬细胞增生并可见吞噬现象；可见脾脏贫血性梗死。淋巴结淋巴细胞数量较少，可见坏死。免疫组化染色显示脾脏和淋巴结内 CD4+ T 和 CD8+T 细胞均减少。淋巴结组织可呈新型冠状病毒核酸检测阳性，巨噬细胞新型冠状病毒抗原免疫染色阳性。骨髓造血细胞或增生或数量减少，粒红比例增高；偶见噬血现象。

（三）心脏和血管。

部分心肌细胞可见变性、坏死，间质充血、水肿，可见少数单核细胞、淋巴细胞和（或）中性粒细胞浸润。偶见新型冠状病毒核酸检测阳性。

全身主要部位小血管可见内皮细胞脱落、内膜或全层炎症；可见血管内混合血栓形成、血栓栓塞及相应部位的梗死。主要脏器微血管可见透明血栓形成。

（四）肝脏和胆囊。

肝细胞变性、灶性坏死伴中性粒细胞浸润；肝血窦充血，汇管区见淋巴细胞和单核细胞细胞浸润，微血栓形成。胆囊高度充盈。肝脏和胆囊可见新型冠状病毒核酸检测阳性。

（五）肾脏。

肾小球毛细血管充血，偶见节段性纤维素样坏死；球囊腔内见蛋白性渗出物。近端小管上皮变性，部分坏死、脱落，远端小管易见管型。肾间质充血，可见微血栓形成。肾组织偶见新型冠状病毒核酸检测阳性。

（六）其他器官。

脑组织充血、水肿，部分神经元变性、缺血性改变和脱失，偶见噬节现象；可见血管周围间隙单核细胞和淋巴细胞浸润。肾上腺见灶性坏死。食管、胃和肠黏膜上皮不同程度变性、坏死、脱落，固有层和黏膜下单核细胞、淋巴细胞浸润。肾上腺可见皮质细胞变性，灶性出血和坏死。睾丸见不同程度的生精细胞数量减少，Sertoli 细胞和 Leydig 细胞变性。

鼻咽和胃肠黏膜及睾丸和唾液腺等器官可检测到新型冠状病毒。

四、临床特点

（一）临床表现。

潜伏期 1～14 天，多为 3～7 天。

以发热、干咳、乏力为主要表现。部分患者以嗅觉、味觉减退或丧失等为首发症状，少数患者伴有鼻塞、流涕、咽痛、结膜炎、肌痛和腹泻等症状。重症患者多在发病一周后出现呼吸困难和（或）低氧血症，严重者可快速进展为急性呼吸窘迫综合征、脓毒症休克、难以纠正的代谢性酸中毒和出凝血功能障碍及多器官功能衰竭等。极少数患者还可有中枢神经系统受累及肢端缺血性坏死等表现。值得注意的是重型、危重型患者病程中可为中低热，甚至无明显发热。

轻型患者可表现为低热、轻微乏力、嗅觉及味觉障碍等，无肺炎表现。少数患者在感染新型冠状病毒后可无明显临床症状。

多数患者预后良好，少数患者病情危重，多见于老年人、有慢性基础疾病者、晚期妊娠和围产期女性、肥胖人群。

儿童病例症状相对较轻，部分儿童及新生儿病例症状可不典型，表现为呕吐、腹泻等消化道症状或仅表现为反应差、呼吸急促。极少数儿童可有多系统炎症综合征（MIS-C），出现类似川崎病或不典型川崎病表现、中毒性休克综合征或巨噬细胞活化综合征等，多发生于恢复期。主要表现为发热伴皮疹、非化脓性结膜炎、黏膜炎症、低血压或休克、凝血障碍、急性消化道症状等。一旦发生，病情可在短期内急剧恶化。

（二）实验室检查。

1. 一般检查

发病早期外周血白细胞总数正常或减少，可见淋巴细胞计数减少，部分患者可出现肝酶、乳酸脱氢酶、肌酶、肌红蛋白、肌钙蛋白和铁蛋白增高。多数患者C反应蛋白（CRP）和血沉升高，降钙素原正常。重型、危重型患者可见D-二聚体升高、外周血淋巴细胞进行性减少，炎症因子升高。

2. 病原学及血清学检查

（1）病原学检查:采用RT-PCR和（或）NGS方法在鼻咽拭子、痰和其他下呼吸道分泌物、血液、粪便、尿液等标本中可检测出新型冠状病毒核酸。检测下呼吸道标本（痰或气道抽取物）更加准确。

核酸检测会受到病程、标本采集、检测过程、检测试剂等因素的影响，为提高检测阳性率，应规范采集标本，标本采集后尽快送检。

（2）血清学检查:新型冠状病毒特异性IgM抗体、IgG抗体阳性，发病1周内阳性率均较低。

由于试剂本身阳性判断值原因，或者体内存在干扰物质（类风湿因子、嗜异性抗体、补体、溶菌酶等），或者标本原因（标本溶血、标本被细菌污染、标本贮存时间过长、标本凝固不全等），抗体检测可能会出现假阳性。一般不单独以血清学检测作为诊断依据，需结合流行病学史、临床表现和基础疾病等情况进行综合判断。

对以下患者可通过抗体检测进行诊断:①临床怀疑新冠肺炎且核酸检测阴性的患者；②病情处于恢复期且核酸检测阴性

的患者。

（三）胸部影像学。

早期呈现多发小斑片影及间质改变，以肺外带明显。进而发展为双肺多发磨玻璃影、浸润影，严重者可出现肺实变，胸腔积液少见。MIS-C 时，心功能不全患者可见心影增大和肺水肿。

五、诊断标准

（一）疑似病例。

结合下述流行病学史和临床表现综合分析，有流行病学史中的任何 1 条，且符合临床表现中任意 2 条。

无明确流行病学史的，符合临床表现中任意 2 条，同时新型冠状病毒特异性 IgM 抗体阳性；或符合临床表现中的 3 条。

1.流行病学史

（1）发病前 14 天内有病例报告社区的旅行史或居住史；

（2）发病前 14 天内与新型冠状病毒感染的患者或无症状感染者有接触史；

（3）发病前 14 天内曾接触过来自有病例报告社区的发热或有呼吸道症状的患者；

（4）聚集性发病（2 周内在小范围如家庭、办公室、学校班级等场所，出现 2 例及以上发热和/或呼吸道症状的病例）。

2.临床表现

（1）发热和（或）呼吸道症状等新冠肺炎相关临床表现；

（2）具有上述新冠肺炎影像学特征；

（3）发病早期白细胞总数正常或降低，淋巴细胞计数正常或减少。

（二）确诊病例。

疑似病例同时具备以下病原学或血清学证据之一者:

1.实时荧光 RT-PCR 检测新型冠状病毒核酸阳性;

2.病毒基因测序，与已知的新型冠状病毒高度同源;

3.新型冠状病毒特异性 IgM 抗体和 IgG 抗体阳性;

4.新型冠状病毒特异性 IgG 抗体由阴性转为阳性或恢复期 IgG 抗体滴度较急性期呈 4 倍及以上升高。

六、临床分型

（一）轻型。

临床症状轻微，影像学未见肺炎表现。

（二）普通型。

具有发热、呼吸道症状等，影像学可见肺炎表现。

（三）重型。

成人符合下列任何一条:

1.出现气促，RR≥30 次/分;

2.静息状态下，吸空气时指氧饱和度≤93%;

3.动脉血氧分压（PaO_2）/吸氧浓度（FiO_2）≤300mmHg（1mmHg=0.133kPa）;

高海拔(海拔超过1000米)地区应根据以下公式对 PaO_2/FiO_2 进行校正: PaO_2/FiO_2×[760/大气压(mmHg)]。

4. 临床症状进行性加重，肺部影像学显示 24～48 小时内病灶明显进展>50%者。

儿童符合下列任何一条:

1.持续高热超过 3 天;

2. 出现气促（<2 月龄，RR≥60 次/分；2～12 月龄，RR≥50 次/分；1～5 岁，RR≥40 次/分；>5 岁，RR≥30 次/分），除外发热和哭闹的影响；

3. 静息状态下，吸空气时指氧饱和度≤93%；

4. 辅助呼吸（鼻翼扇动、三凹征）；

5. 出现嗜睡、惊厥；

6. 拒食或喂养困难，有脱水征。

（四）危重型。

符合以下情况之一者：

1. 出现呼吸衰竭，且需要机械通气；

2. 出现休克；

3. 合并其他器官功能衰竭需 ICU 监护治疗。

七、重型/危重型高危人群

（一）大于 65 岁老年人；

（二）有心脑血管疾病（含高血压）、慢性肺部疾病（慢性阻塞性肺疾病、中度至重度哮喘）、糖尿病、慢性肝脏、肾脏疾病、肿瘤等基础疾病者；

（三）免疫功能缺陷（如艾滋病患者、长期使用皮质类固醇或其他免疫抑制药物导致免疫功能减退状态）；

（四）肥胖（体质指数≥30）；

（五）晚期妊娠和围产期女性；

（六）重度吸烟者。

八、重型/危重型早期预警指标

（一）成人。

有以下指标变化应警惕病情恶化：

1.低氧血症或呼吸窘迫进行性加重；

2.组织氧合指标恶化或乳酸进行性升高；

3.外周血淋巴细胞计数进行性降低或外周血炎症标记物如IL-6、CRP、铁蛋白等进行性上升；

4.D-二聚体等凝血功能相关指标明显升高；

5.胸部影像学显示肺部病变明显进展。

（二）儿童。

1.呼吸频率增快；

2.精神反应差、嗜睡；

3.乳酸进行性升高；

4.CRP、PCT、铁蛋白等炎症标记物明显升高；

5.影像学显示双侧或多肺叶浸润、胸腔积液或短期内病变快速进展；

6.有基础疾病（先天性心脏病、支气管肺发育不良、呼吸道畸形、异常血红蛋白、重度营养不良等）、有免疫缺陷或低下（长期使用免疫抑制剂）和新生儿。

九、鉴别诊断

（一）新型冠状病毒肺炎轻型表现需与其它病毒引起的上呼吸道感染相鉴别。

（二）新型冠状病毒肺炎主要与流感病毒、腺病毒、呼吸道合胞病毒等其他已知病毒性肺炎及肺炎支原体感染鉴别，尤其是对疑似病例要尽可能采取包括快速抗原检测和多重PCR核酸检测等方法，对常见呼吸道病原体进行检测。

（三）还要与非感染性疾病，如血管炎、皮肌炎和机化性肺炎等鉴别。

（四）儿童患者出现皮疹、黏膜损害时，需与川崎病鉴别。

十、病例的发现与报告

各级各类医疗机构的医务人员发现符合病例定义的疑似病例后，应当立即进行单人单间隔离治疗，院内专家会诊或主诊医师会诊，仍考虑疑似病例，在 2 小时内进行网络直报，并采集标本进行新型冠状病毒核酸检测，同时在确保转运安全前提下立即将疑似病例转运至定点医院。与新型冠状病毒感染者有密切接触者，即便常见呼吸道病原检测阳性，也建议及时进行新型冠状病毒病原学检测。疑似病例连续两次新型冠状病毒核酸检测阴性（采样时间至少间隔 24 小时）且发病 7 天后新型冠状病毒特异性 IgM 抗体和 IgG 抗体仍为阴性可排除疑似病例诊断。

对于确诊病例应在发现后 2 小时内进行网络直报。

十一、治疗

（一）根据病情确定治疗场所。

1.疑似及确诊病例应在具备有效隔离条件和防护条件的定点医院隔离治疗，疑似病例应单人单间隔离治疗，确诊病例可多人收治在同一病室。

2.危重型病例应当尽早收入 ICU 治疗。

（二）一般治疗。

1.卧床休息，加强支持治疗，保证充分能量摄入；注意水、电解质平衡，维持内环境稳定；密切监测生命体征、指氧饱和度等。

2.根据病情监测血常规、尿常规、CRP、生化指标（肝酶、心肌酶、肾功能等）、凝血功能、动脉血气分析、胸部影像学等。有条件者可行细胞因子检测。

3.及时给予有效氧疗措施，包括鼻导管、面罩给氧和经鼻高流量氧疗。有条件可采用氢氧混合吸入气（H_2/O_2: 66.6%/33.3%）治疗。

4.抗菌药物治疗:避免盲目或不恰当使用抗菌药物，尤其是联合使用广谱抗菌药物。

（三）抗病毒治疗。

在抗病毒药物应急性临床试用过程中，相继开展了多项临床试验，虽然仍未发现经严格“随机、双盲、安慰剂对照研究”证明有效的抗病毒药物，但某些药物经临床观察研究显示可能具有一定的治疗作用。目前较为一致的意见认为，具有潜在抗病毒作用的药物应在病程早期使用，建议重点应用于有重症高危因素及有重症倾向的患者。

不推荐单独使用洛匹那韦/利托那韦和利巴韦林，不推荐使用羟氯喹或联合使用阿奇霉素。以下药物可继续试用，在临床应用中进一步评价疗效。

1.α-干扰素:成人每次 500 万 U 或相当剂量，加入灭菌注射用水 2ml，每日 2 次，雾化吸入，疗程不超过 10 天;

2.利巴韦林:建议与干扰素（剂量同上）或洛匹那韦/利托那韦（成人 200mg/50mg/粒，每次 2 粒，每日 2 次）联合应用，成人 500mg/次，每日 2 至 3 次静脉输注，疗程不超过 10 天;

3.磷酸氯喹:用于 18 岁～65 岁成人。体重大于 50kg 者，每次 500mg，每日 2 次，疗程 7 天;体重小于 50kg 者，第 1、2 天每次 500mg，每日 2 次，第 3～7 天每次 500mg，每日 1 次;

4.阿比多尔:成人 200mg，每日 3 次，疗程不超过 10 天。

要注意上述药物的不良反应、禁忌症以及与其他药物的相互作用等问题。不建议同时应用 3 种以上抗病毒药物，出现不可耐受的毒副作用时应停止使用相关药物。对孕产妇患者的治疗应考虑妊娠周数，尽可能选择对胎儿影响较小的药物，以及考虑是否终止妊娠后再进行治疗，并知情告知。

（四）免疫治疗。

1.康复者恢复期血浆:适用于病情进展较快、重型和危重型患者。用法用量参考《新冠肺炎康复者恢复期血浆临床治疗方案（试行第二版)》。

2.静注 COVID-19 人免疫球蛋白:可应急用于病情进展较快的普通型和重型患者。推荐剂量为普通型 20ml、重型 40ml，静脉输注，根据患者病情改善情况，可隔日再次输注，总次数不超过 5 次。

3.托珠单抗:对于双肺广泛病变者及重型患者，且实验室检测 IL-6 水平升高者，可试用。具体用法:首次剂量 4～8mg/kg，推荐剂量 400mg，0.9%生理盐水稀释至 100ml，输注时间大于 1 小时;首次用药疗效不佳者，可在首剂应用 12 小时后追加应用

一次（剂量同前），累计给药次数最多为2次，单次最大剂量不超过800mg。注意过敏反应，有结核等活动性感染者禁用。

（五）糖皮质激素治疗。

对于氧合指标进行性恶化、影像学进展迅速、机体炎症反应过度激活状态的患者，酌情短期内（一般建议3～5日，不超过10日）使用糖皮质激素，建议剂量相当于甲泼尼龙0.5～1mg/kg/日，应当注意较大剂量糖皮质激素由于免疫抑制作用，可能会延缓对病毒的清除。

（六）重型、危重型病例的治疗。

1.治疗原则:在上述治疗的基础上，积极防治并发症，治疗基础疾病，预防继发感染，及时进行器官功能支持。

2.呼吸支持:

（1）鼻导管或面罩吸氧

PaO_2/FiO_2低于300 mmHg的重型患者均应立即给予氧疗。接受鼻导管或面罩吸氧后，短时间（1～2小时）密切观察，若呼吸窘迫和(或)低氧血症无改善，应使用经鼻高流量氧疗(HFNC)或无创通气（NIV)。

（2）经鼻高流量氧疗或无创通气

PaO_2/FiO_2低于200 mmHg应给予经鼻高流量氧疗（HFNC）或无创通气(NIV)。接受HFNC或NIV的患者，无禁忌症的情况下，建议同时实施俯卧位通气，即清醒俯卧位通气，俯卧位治疗时间应大于12小时。

部分患者使用HFNC或NIV治疗的失败风险高，需要密切观察患者的症状和体征。若短时间（1～2小时）治疗后病情无改

善，特别是接受俯卧位治疗后，低氧血症仍无改善，或呼吸频数、潮气量过大或吸气努力过强等，往往提示 HFNC 或 NIV 治疗疗效不佳，应及时进行有创机械通气治疗。

（3）有创机械通气

一般情况下，PaO_2/FiO_2低于 150 mmHg，应考虑气管插管，实施有创机械通气。但鉴于重症新型冠状病毒肺炎患者低氧血症的临床表现不典型，不应单纯把PaO_2/FiO_2是否达标作为气管插管和有创机械通气的指征，而应结合患者的临床表现和器官功能情况实时进行评估。值得注意的是，延误气管插管，带来的危害可能更大。

早期恰当的有创机械通气治疗是危重型患者重要的治疗手段。实施肺保护性机械通气策略。对于中重度急性呼吸窘迫综合征患者,或有创机械通气 FiO_2高于 50%时,可采用肺复张治疗。并根据肺复张的反应性，决定是否反复实施肺复张手法。应注意部分新冠肺炎患者肺可复张性较差，应避免过高的 PEEP 导致气压伤。

（4）气道管理

加强气道湿化，建议采用主动加热湿化器，有条件的使用环路加热导丝保证湿化效果；建议使用密闭式吸痰，必要时气管镜吸痰；积极进行气道廓清治疗，如振动排痰、高频胸廓振荡、体位引流等；在氧合及血流动力学稳定的情况下，尽早开展被动及主动活动，促进痰液引流及肺康复。

（5）体外膜肺氧合（ECMO）

ECMO 启动时机。在最优的机械通气条件下（FiO_2≥80%，潮气量为 6 ml/kg 理想体重，PEEP≥5 cmH_2O，且无禁忌症），且保护性通气和俯卧位通气效果不佳，并符合以下之一，应尽早考虑评估实施 ECMO:

① PaO_2/FiO_2＜50 mmHg 超过 3 小时;

② PaO_2/FiO_2＜80 mmHg 超过 6 小时;

③ 动脉血 pH＜7.25 且 $PaCO_2$＞60 mmHg 超过 6 小时，且呼吸频率＞35 次/分;

④ 呼吸频率＞35 次/分时，动脉血 pH＜7.2 且平台压>30 cmH_2O;

⑤ 合并心源性休克或者心脏骤停。

符合ECMO指征,且无禁忌症的危重型患者,应尽早启动ECMO治疗，延误时机，导致患者预后不良。

ECMO 模式选择。仅需呼吸支持时选用静脉-静脉方式 ECMO（VV- ECMO），是最为常用的方式；需呼吸和循环同时支持则选用静脉-动脉方式 ECMO（VA-ECMO）；VA-ECMO 出现头臂部缺氧时可采用 VAV-ECMO 模式。实施 ECMO 后，严格实施肺保护性肺通气策略。推荐初始设置:潮气量＜4～6ml/Kg 理想体重，平台压≤25cmH_2O，驱动压＜15cmH_2O，PEEP 5～15cmH_2O，呼吸频率 4～10 次/分，FiO_2＜50%。对于氧合功能难以维持或吸气努力强、双肺重力依赖区实变明显、或需积极气道分泌物引流的患者，可联合俯卧位通气。

儿童心肺代偿能力较成人弱，对缺氧更为敏感，需要应用比成人更积极的氧疗和通气支持策略，指征应适当放宽；不推

荐常规应用肺复张。

3.循环支持:危重型患者可合并休克，应在充分液体复苏的基础上，合理使用血管活性药物，密切监测患者血压、心率和尿量的变化，以及乳酸和碱剩余。必要时进行血流动力学监测，指导输液和血管活性药物使用，改善组织灌注。

4.抗凝治疗:重型或危重型患者合并血栓栓塞风险较高。对无抗凝禁忌症者，同时D-二聚体明显增高者，建议预防性使用抗凝药物。发生血栓栓塞事件时，按照相应指南进行抗凝治疗。

5.急性肾损伤和肾替代治疗:危重型患者可合并急性肾损伤，应积极寻找病因，如低灌注和药物等因素。在积极纠正病因的同时，注意维持水、电解质、酸碱平衡。连续性肾替代治疗（CRRT）的指征包括:①高钾血症；②严重酸中毒；③利尿剂无效的肺水肿或水负荷过多。

6.血液净化治疗:血液净化系统包括血浆置换、吸附、灌流、血液/血浆滤过等，能清除炎症因子，阻断“细胞因子风暴”，从而减轻炎症反应对机体的损伤，可用于重型、危重型患者细胞因子风暴早中期的救治。

7.儿童多系统炎症综合征:治疗原则是多学科合作，尽早抗炎、纠正休克和出凝血功能障碍、脏器功能支持，必要时抗感染治疗。有典型或不典型川崎病表现者，与川崎病经典治疗方案相似。以静脉用丙种球蛋白（IVIG）、糖皮质激素及口服阿司匹林等治疗为主。

8.其他治疗措施可考虑使用血必净治疗；可使用肠道微生态调节剂，维持肠道微生态平衡，预防继发细菌感染；儿童重

型、危重型病例可酌情考虑使用 IVIG。

妊娠合并重型或危重型患者应积极终止妊娠，剖腹产为首选。

患者常存在焦虑恐惧情绪，应当加强心理疏导，必要时辅以药物治疗。

（七）中医治疗。

本病属于中医“疫”病范畴，病因为感受“疫戾”之气，各地可根据病情、当地气候特点以及不同体质等情况，参照下列方案进行辨证论治。涉及到超药典剂量，应当在医师指导下使用。

1.医学观察期

临床表现 1: 乏力伴胃肠不适

推荐中成药: 藿香正气胶囊（丸、水、口服液）

临床表现 2: 乏力伴发热

推荐中成药: 金花清感颗粒、连花清瘟胶囊（颗粒）、疏风解毒胶囊（颗粒）

2.临床治疗期（确诊病例）

2.1 清肺排毒汤

适用范围: 结合多地医生临床观察，**适用于轻型、普通型、重型患者，在危重型患者救治中可结合患者实际情况合理使用。**

基础方剂: 麻黄 9g、炙甘草 6g、杏仁 9g、生石膏 15～30g（先煎）、桂枝 9g、泽泻 9g、猪苓 9g、白术 9g、茯苓 15g、柴胡 16g、黄芩 6g、姜半夏 9g、生姜 9g、紫菀 9g、冬花 9g、射干 9g、细辛 6g、山药 12g、枳实 6g、陈皮 6g、藿香 9g。

服法: 传统中药饮片，水煎服。每天一付，早晚各一次（饭后四十分钟），温服，三付一个疗程。

如有条件，每次服完药可加服大米汤半碗，舌干津液亏虚者可多服至一碗。（注：如患者不发热则生石膏的用量要小，发热或壮热可加大生石膏用量）。若症状好转而未痊愈则服用第二个疗程，若患者有特殊情况或其他基础病，第二疗程可以根据实际情况修改处方，症状消失则停药。

处方来源: 国家卫生健康委办公厅 国家中医药管理局办公室《关于推荐在中西医结合救治新型冠状病毒感染的肺炎中使用“清肺排毒汤”的通知》(国中医药办医政函〔2020〕22 号)。

2.2 轻型

(1) 寒湿郁肺证

临床表现: 发热，乏力，周身酸痛，咳嗽，咯痰，胸紧憋气，纳呆，恶心，呕吐，大便粘腻不爽。舌质淡胖齿痕或淡红,苔白厚腐腻或白腻，脉濡或滑。

推荐处方: 寒湿疫方

基础方剂: 生麻黄 6g、生石膏 15g、杏仁 9g、羌活 15g、葶苈子 15g、贯众 9g、地龙 15g、徐长卿 15g、藿香 15g、佩兰 9g、苍术 15g、云苓 45g、生白术 30g、焦三仙各 9g、厚朴 15g、焦槟榔 9g、煨草果 9g、生姜 15g。

服法: 每日 1 剂,水煎 600ml,分 3 次服用,早中晚各 1 次，饭前服用。

(2) 湿热蕴肺证

临床表现: 低热或不发热，微恶寒，乏力，头身困重，肌肉

酸痛，干咳痰少，咽痛，口干不欲多饮，或伴有胸闷脘痞，无汗或汗出不畅，或见呕恶纳呆，便溏或大便粘滞不爽。舌淡红，苔白厚腻或薄黄，脉滑数或濡。

推荐处方: 槟榔 10g、草果 10g、厚朴 10g、知母 10g、黄芩 10g、柴胡 10g、赤芍 10g、连翘 15g、青蒿 10g（后下）、苍术 10g、大青叶 10g、生甘草 5g。

服法: 每日 1 剂，水煎 400ml,分 2 次服用，早晚各 1 次。

2.3 普通型

(1)湿毒郁肺证

临床表现: 发热，咳嗽痰少，或有黄痰，憋闷气促，腹胀，便秘不畅。舌质暗红，舌体胖，苔黄腻或黄燥，脉滑数或弦滑。

推荐处方: 宣肺败毒方

基础方剂: 生麻黄 6g、苦杏仁 15g、生石膏 30g、生薏苡仁 30g、茅苍术 10g、广藿香 15g、青蒿草 12g、虎杖 20g、马鞭草 30g、干芦根 30g、葶苈子 15g、化橘红 15g、生甘草 10g。

服法: 每日 1 剂，水煎 400ml,分 2 次服用，早晚各 1 次。

(2)寒湿阻肺证

临床表现: 低热，身热不扬，或未热，干咳，少痰，倦怠乏力，胸闷，脘痞，或呕恶，便溏。舌质淡或淡红，苔白或白腻，脉濡。

推荐处方: 苍术 15g、陈皮 10g、厚朴 10g、藿香 10g、草果 6g、生麻黄 6g、羌活 10g、生姜 10g、槟榔 10g。

服法: 每日 1 剂，水煎 400ml,分 2 次服用，早晚各 1 次。

2.4 重型

(1)疫毒闭肺证

临床表现: 发热面红，咳嗽，痰黄粘少，或痰中带血，喘憋气促，疲乏倦怠，口干苦粘，恶心不食，大便不畅，小便短赤。舌红，苔黄腻，脉滑数。

推荐处方: 化湿败毒方

基础方剂: 生麻黄 6g、杏仁 9g、生石膏 15g、甘草 3g、藿香 10g （后下）、厚朴 10g、苍术 15g、草果 10g、法半夏 9g、茯苓 15g、生大黄 5g （后下）、生黄芪 10g、葶苈子 10g、赤芍 10g。

服法: 每日 1～2 剂，水煎服，每次 100ml～200ml，一日 2～4 次，口服或鼻饲。

（2）气营两燔证

临床表现: 大热烦渴，喘憋气促，谵语神昏，视物错瞀，或发斑疹，或吐血、衄血，或四肢抽搐。舌绛少苔或无苔，脉沉细数，或浮大而数。

推荐处方: 生石膏 30～60g （先煎）、知母 30g、生地 30～60g、水牛角 30g （先煎）、赤芍 30g、玄参 30g、连翘 15g、丹皮 15g、黄连 6g、竹叶 12g、葶苈子 15g、生甘草 6g。

服法: 每日 1 剂，水煎服，先煎石膏、水牛角后下诸药，每次 100ml～200ml，每日 2～4 次，口服或鼻饲。

推荐中成药: 喜炎平注射液、血必净注射液、热毒宁注射液、痰热清注射液、醒脑静注射液。功效相近的药物根据个体情况可选择一种，也可根据临床症状联合使用两种。中药注射剂可与中药汤剂联合使用。

2.5 危重型

内闭外脱证

临床表现: 呼吸困难、动辄气喘或需要机械通气，伴神昏，烦躁，汗出肢冷，舌质紫暗，苔厚腻或燥，脉浮大无根。

推荐处方: 人参 15g、黑顺片 10g（先煎）、山茱萸 15g,送服苏合香丸或安宫牛黄丸。

出现机械通气伴腹胀便秘或大便不畅者，可用生大黄 5～10g。出现人机不同步情况，在镇静和肌松剂使用的情况下，可用生大黄 5～10g 和芒硝 5～10g。

推荐中成药: 血必净注射液、热毒宁注射液、痰热清注射液、醒脑静注射液、参附注射液、生脉注射液、参麦注射液。功效相近的药物根据个体情况可选择一种，也可根据临床症状联合使用两种。中药注射剂可与中药汤剂联合使用。

注:重型和危重型中药注射剂推荐用法

中药注射剂的使用遵照药品说明书从小剂量开始、逐步辨证调整的原则，推荐用法如下:

病毒感染或合并轻度细菌感染: 0.9%氯化钠注射液 250ml 加喜炎平注射液 100mg bid,或 0.9%氯化钠注射液 250ml 加热毒宁注射液 20ml,或 0.9%氯化钠注射液 250ml 加痰热清注射液 40ml bid。

高热伴意识障碍: 0.9%氯化钠注射液 250ml 加醒脑静注射液 20ml bid。

全身炎症反应综合征或/和多脏器功能衰竭: 0.9%氯化钠注射液 250ml 加血必净注射液 100ml bid。

免疫抑制: 葡萄糖注射液 250ml 加参麦注射液 100ml 或生脉注射液 20～60ml bid。

2.6 恢复期

（1） 肺脾气虚证

临床表现: 气短，倦怠乏力，纳差呕恶，痞满，大便无力，便溏不爽。舌淡胖，苔白腻。

推荐处方: 法半夏 9g、陈皮 10g、党参 15g、炙黄芪 30g、 炒白术 10g、茯苓 15g、藿香 10g、砂仁 6g （后下）、甘草 6g。

服法: 每日 1 剂，水煎 400ml,分 2 次服用，早晚各 1 次。

（2） 气阴两虚证

临床表现: 乏力，气短，口干，口渴，心悸，汗多，纳差，低热或不热，干咳少痰。舌干少津，脉细或虚无力。

推荐处方: 南北沙参各 10g、麦冬 15g、西洋参 6g,五味子 6g、生石膏 15g、淡竹叶 10g、桑叶 10g、芦根 15g、丹参 15g、生甘草 6g。

服法: 每日 1 剂，水煎 400ml,分 2 次服用，早晚各 1 次。

（八）早期康复

重视患者早期康复介入，针对新冠肺炎患者呼吸功能、躯体功能以及心理障碍，积极开展康复训练和干预，尽最大可能恢复体能、体质和免疫能力。

十二、护理

根据患者病情，明确护理重点并做好基础护理。重症患者密切观察患者生命体征和意识状态，重点监测血氧饱和度。危重症患者 24 小时持续心电监测，每小时测量患者的心率、呼吸

频率、血压、SpO_2，每 4 小时测量并记录体温。合理、正确使用静脉通路，并保持各类管路通畅，妥善固定。卧床患者定时变更体位，预防压力性损伤。按护理规范做好无创机械通气、有创机械通气、人工气道、俯卧位通气、镇静镇痛、体外膜肺氧合诊疗的护理。特别注意患者口腔护理和液体出入量管理，有创机械通气患者防止误吸。清醒患者及时评估心理状况，做好心理护理。

十三、出院标准及出院后注意事项

（一）出院标准。

1. 体温恢复正常 3 天以上;

2. 呼吸道症状明显好转;

3. 肺部影像学显示急性渗出性病变明显改善;

4. 连续两次呼吸道标本核酸检测阴性（采样时间至少间隔 24 小时）。

满足以上条件者可出院。

对于满足上述第 1、2、3 条标准的患者，核酸仍持续阳性超过 4 周者，建议通过抗体检测、病毒培养分离等方法对患者传染性进行综合评估后，判断是否出院。

（二）出院后注意事项。

1. 定点医院要做好与患者居住地基层医疗机构间的联系，共享病历资料，及时将出院患者信息推送至患者辖区或居住地基层医疗卫生机构。

2. 建议出院后继续进行 14 天隔离管理和健康状况监测，佩戴口罩，有条件的居住在通风良好的单人房间，减少与家人的

近距离密切接触，分餐饮食，做好手卫生，避免外出活动。

3.建议在出院后第2周、第4周到医院随访、复诊。

十四、转运原则

按照国家卫生健康委印发的《新型冠状病毒感染的肺炎病例转运工作方案（试行）》执行。

十五、医疗机构内感染预防与控制

严格按照国家卫生健康委印发的《医疗机构内新型冠状病毒感染预防与控制技术指南（第一版）》、《新型冠状病毒感染的肺炎防护中常见医用防护用品使用范围指引（试行）》的要求执行。

十六、预防

保持良好的个人及环境卫生，均衡营养、适量运动、充足休息，避免过度疲劳。提高健康素养，养成“一米线”、勤洗手、戴口罩、公筷制等卫生习惯和生活方式，打喷嚏或咳嗽时应掩住口鼻。保持室内通风良好，科学做好个人防护，出现呼吸道症状时应及时到发热门诊就医。近期去过高风险地区或与确诊、疑似病例有接触史的，应主动进行新型冠状病毒核酸检测。
